# Sustainable production of valuable compound 3-succinoyl-pyridine by genetically engineering *Pseudomonas putida* using the tobacco waste

**DOI:** 10.1038/srep16411

**Published:** 2015-11-17

**Authors:** Weiwei Wang, Ping Xu, Hongzhi Tang

**Affiliations:** 1 State Key Laboratory of Microbial Metabolism, and School of Life Sciences & Biotechnology, Shanghai Jiao Tong University, Shanghai 200240, People’s Republic of China

## Abstract

Treatment of solid and liquid tobacco wastes with high nicotine content remains a longstanding challenge. Here, we explored an environmentally friendly approach to replace tobacco waste disposal with resource recovery by genetically engineering *Pseudomonas putida*. The biosynthesis of 3-succinoyl-pyridine (SP), a precursor in the production of hypotensive agents, from the tobacco waste was developed using whole cells of the engineered *Pseudomonas* strain, S16dspm. Under optimal conditions in fed-batch biotransformation, the final concentrations of product SP reached 9.8 g/L and 8.9 g/L from aqueous nicotine solution and crude suspension of the tobacco waste, respectively. In addition, the crystal compound SP produced from aqueous nicotine of the tobacco waste in batch biotransformation was of high purity and its isolation yield on nicotine was 54.2%. This study shows a promising route for processing environmental wastes as raw materials in order to produce valuable compounds.

Tobacco (*Nicotiana*, of the Solanaceae family), cash crop with a long history, is widely cultivated in USA, China, India, Brazil, and Cuba. China is the largest producer and consumer of tobacco worldwide, accounting for about a third of the total global consumption^1^. However, parts of tobacco leaves that can not be used in cigarette production are discarded because their nicotine content is in a high range of 3–6% (w/w)^2^. As an *N*-heterocyclic alkaloid, nicotine is the main toxic organic compound in solid or liquid tobacco wastes. The lethal dose of pure nicotine for adults is reported to be 60 mg, an oral LD_50_ of 0.8 mg/kg, or less (30–60 mg)^3^. In addition, nicotine released into the environment as a result of the tobacco industry has been designated as a Toxic Release Inventory (TRI) chemical by the United States Environmental Protection Agency^4^. Due to its high solubility in aqueous solutions, nicotine leached from tobacco wastes can be easily transported into ground water. In previous reports, nicotine has been increasingly detected in seepage and leakage water from landfill wastes^5^,^6^.

The development of methods to clean up and remove nicotine present in large amounts of tobacco wastes is imperative. Under methanogenic or aerobic conditions, a series of effective bioremediation processes focusing on nicotine reduction in tobacco wastes have been developed^7^,^8^,^9^. However, a variety of microorganisms play important roles in these biological methods. Bacteria of the genera *Pseudomonas* and *Arthrobacter* represent the two major types of bacterial species among these nicotinophilic microorganisms^10^. Because of their ability to degrade nicotine with high efficiency, certain microbes could be used to dispose of tobacco wastes^11^. Replacing waste disposal with resource recovery has been viewed as a potential environment-friendly and resource-saving future possibility^12^. In earlier times, to increase the value of tobacco wastes, such as low-grade tobacco leaves, processes were carried out to recover nicotine from these wastes and the extracted nicotine or nicotine sulfate was used as insecticide^13^,^14^,^15^. In addition, nicotine has potential application as a precursor molecule for the synthesis of valuable chemicals, especially many functional pyridines, which are difficult to prepare via chemical methods^16^,^17^. A number of pyridine derivatives exhibit bioactivity and medicinal value, such as vitamin B_6_ (pyridoxine), analgesics (propiram, epibatidine), analeptics (nicethamide, camphotamide) and anti-inflammaroty drugs (clonixin, nicoboxil) (Fig. 1)^17^,^18^,^19^. For example, 3-succinoyl-pyridine (SP) (also called *γ*-oxo-3-pyridinebutanoic acid) is a nicotine derivative and simple molecular pyridine compound and can be transformed into mammals hypotensive agents (ω-heteroaroyl-(propionyl)-L-prolines)^20^. The mechanism of nicotine degradation in species of the *Arthrobacter* genus has been reported in detail^21^. Moreover, the nicotine degradation pathway in *Pseudomonas* spp. has also been determined, particularly in *Pseudomonas putida* strain S16^22^,^23^,^24^,^25^. Based on these scientific developments, metabolic engineering could be a new approach for realizing customized multistep microbial synthesis of valuable compounds^26^. In previous studies, we have shown that strain S16 and its derivative have the ability to produce 6-hydroxy-3-succinoyl-pyridine (HSP)^13^,^27^, and these results verify that these species of *Pseudomonas* have the potential to utilize the nicotine in tobacco wastes and form a variety of valuable metabolites.

In this study, we expected to introduce a promising “green” method of reusing and reducing the toxicity of tobacco wastes to efficiently synthesize SP using genetically engineering *Pseudomonas putida*. Moreover, this SP production could be initiated with aqueous nicotine solution extracted from the low-grade tobacco leaves and even begun directly with crude suspension of tobacco leaf powder. Therefore, this green strategy makes it possible to convert nicotine from tobacco wastes with high nicotine content into commercially valuable compounds.

## Results

### Detection of nicotine in discarded tobacco leaves

To determine the actual nicotine content of discarded tobacco leaves (the tobacco waste), linear standard curves of nicotine and 3-succinoyl-pyridine (SP) were drawn according to the data of high performance liquid chromatography (HPLC) analysis. Using the standard curve, the nicotine content in these tobacco leaves was calculated as a percentage, yielding results of 3.09% ± 0.02% (w/w) (Table 1). This value was used to determine the total amount of nicotine in the tobacco leaves.

### Separation of nicotine from the tobacco leaves

**Figure 2:**
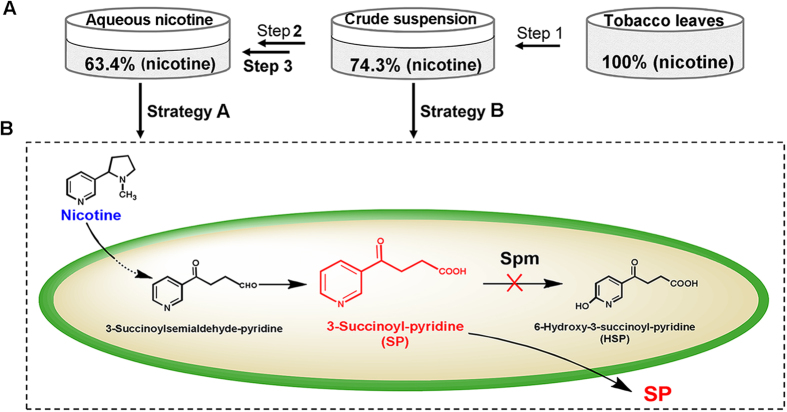
Strategies of SP production from the tobacco leaves using *P. putida* S16dspm. (**A**) Step 1: pure water was used as solvent to obtain crude suspension of the tobacco leaves with nicotine recovery yield of 74.3%. Steps 2 and 3: chloroform extraction and acidic aqueous back-extraction were performed to obtain aqueous nicotine solution with nicotine recovery yield of 64.3%. (**B**) Nicotine degradation pathway in *P. putida* S16dspm was blocked at SP due to inactivation of Spm enzyme. Whole cells of S16dspm were used as biocatalysts for SP production following two strategies: the biotransformation to SP from aqueous nicotine solution (strategy **A**) and from crude suspension of the tobacco leaves (strategy **B**).

After the first step from 50 g tobacco leaves powder, 500 mL leach liquor was obtained and the nicotine content was calculated to be 2.3 g/L. The nicotine yield of step 1 was ~74.3% after immersion in water (Fig. 2). The second step was chloroform extraction, and the thin layer chromatography (TLC) results showed that there was little nicotine residue in aqueous phase after extraction. Finally, nicotine was separated into the aqueous sulfuric acid solution, resulting in 20 ml aqueous solution with 49.0 g/L nicotine content and a 63.4% yield by weight of the total nicotine (Fig. 2).

### Preparation of the S16dspm biocatalyst

**Figure 3:**
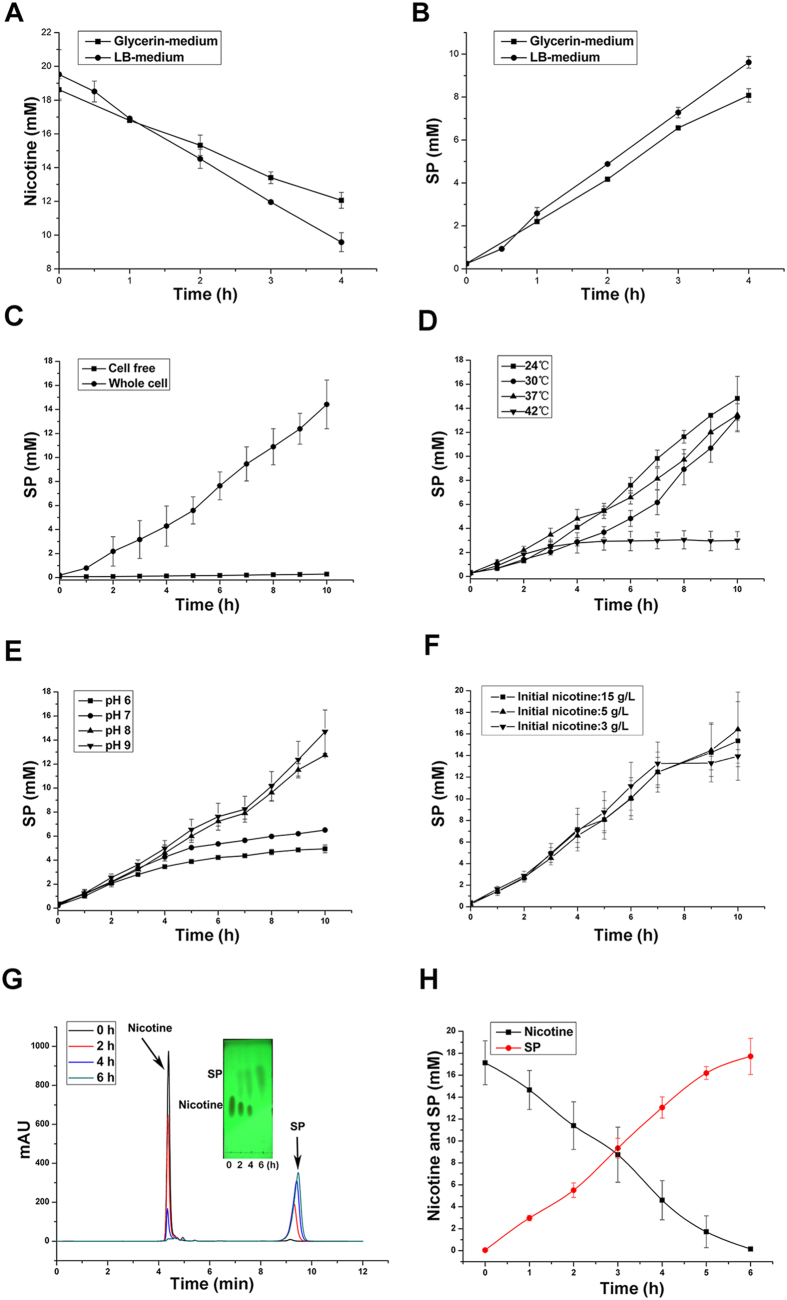
Biotransformation to SP by strain S16dspm, preparation of biocatalyst, and optimal conditions for biotransformation. Preparation of biocatalysts using glycerin (■) and LB (●) medium, respectively. (**A**) Nicotine degradation in the initial stage of resting-cell reactions with glycerin and LB medium. (**B**) SP formation in the initial stage of resting-cell reactions with glycerin and LB medium. Effects of reaction conditions for SP production. (**C**) SP formation by strain S16dspm under whole-cell condition (●) and cell-free condition (■). (**D)** SP formation by strain S16dspm at various temperatures, 24 °C (■), 30 °C (●), 37 °C (▲), 42 °C (▼). (**E**) SP formation by strain S16dspm at various pH values, pH6 (■), pH7 (●), pH8 (▲), pH9 (▼). (**F**) SP formation by strain S16dspm with different initial nicotine contents, 15 g/L (■), 5 g/L (▲), 3 g/L (▼). Analysis of the biotransformation results at the optimal conditions using resting cells of S16dapm. (**G**) TLC and HPLC qualitative analysis of nicotine degradation and SP formation. (**H**) The content changes of nicotine and SP during the biotransformation process detected by HPLC quantitative analysis. Nicotine (■); SP (●).

Due to the deletion of the *spmA* gene, the S16dspm strain has lost the ability to further degrade SP and cannot grow in nicotine medium^25^. Resting-cell reactions of S16dspm were investigated in glycerin and lysogeny borth (LB) medium, respectively. The resting cells obtained from culture in glycerin and LB medium showed no significant difference in nicotine transformation ability (Figs 3A,B). These data suggest that there were no differences between glycerin and LB medium for biocatalyst preparation. However, the whole-cell reactions showed low nicotine cell transforming activity without nicotine in either glycerin or LB medium, which indicated that the nicotine in the prepared media was necessary to improve the bioconversion ability of whole cells as an inducer.

### Optimal reaction conditions for synthesis of SP

Cell integrity was necessary for biotransformation of nicotine to SP because no SP synthesis occurred under cell-free condition (Fig. 3C). Moreover, the nicotine content was detected after 10 h under the cell-free condition, and the nicotine content remained unchanged. Therefore, the biocatalytic reaction should be conducted in the whole-cell system, and cell integrity is necessary for the biotransformation of nicotine.

The biotransformation reactions were performed at different temperatures and pH values in deionized water. The SP synthesis activity was optimal at 24 °C, and the relatively high temperature of 42 °C deteriorated the efficiency of SP formation (Fig. 3D). The acidic condition was found to hinder SP formation, and the optimum pH value was 9.0 (Fig. 3E). According to an earlier report, a nicotine content of 6 g/L or more in culture was poisonous to the growth of bacterium S16^22^. However, under the catalytic reaction condition containing 5.6 mg/ml (dry cell weight, DCW) of resting cells (OD_620_ ~ 10), the biotransformation could be conducted even with an initial nicotine of 15 g/L. Moreover, the SP formation rate was not obviously effected when the initial nicotine content was increased (Fig. 3F). The results suggest that a nicotine content of 3 g/L is an appropriate initial nicotine concentration, and increased substrate concentration could improve the final yield of SP but not the rate of SP formation.

Then, the whole-cell reaction was performed with all of the optimal conditions mentioned above, and the results of TLC/HPLC analysis are shown in Fig. 3G. After incubation for 6 h, almost all of the nicotine has been converted into metabolite SP, and the transformation was an equimolar reaction (Fig. 3H).

### Isolation and identification of the SP compound

**Figure 4:**
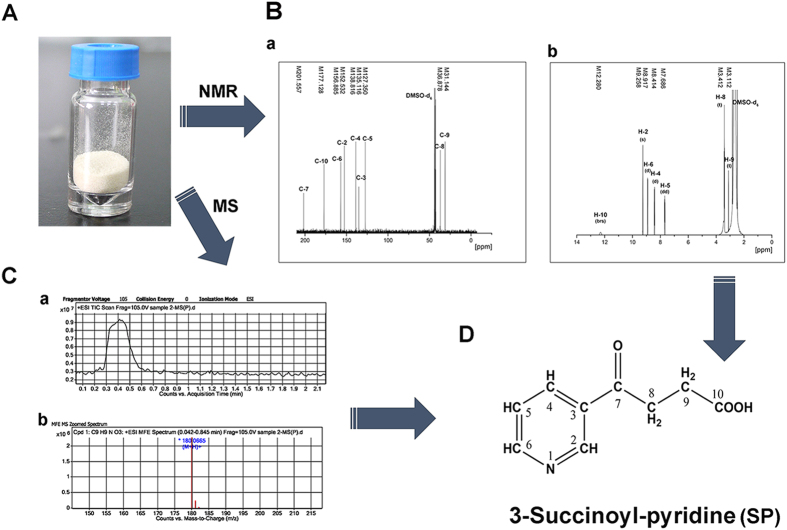
Identification of product SP by ESI-MS and NMR. (**A**) The crystal compound SP got in this study. (**B**) ^13^C-NMR (**B-a**) and ^1^H-NMR (**B-b**) spectra of the product SP. Measurements were performed in DMSO-*d*_*6*_. **C.** The analysis results by ESI-MS. The total ionization chromatography (**C-a**) and the ion mass of [M+H]^+^ (**C-b**) of the product SP. (**D**) Molecular structural formula of SP with the positions of carbon (**C**) and hydrogen (**H**) atoms detected by NMR.

To isolate the metabolite SP, the methods used were performed as previous described and the powder was purified by recrystallization (Fig. 4A)^13^. The crystals showed no impurities when analyzed by HPLC. Finally, about 0.72 g of the (4.02 mmol) metabolite SP was obtained from 1.20 g (7.41 mmol) nicotine and the isolation yield of crystal SP was 54.2%. The structure of metabolite SP was detected and further analyzed by means of mass spectrometry (MS) (*m*/*z* 180.0665 [M + H]^+^) (Fig. 4C) and ^1^H nuclear magnetic resonance (NMR) (DMSO-d_6_, 400 MHz, *δ*: 12.28, 9.26, 8.92, 8.41, 7.69, 3.41, 3.11)/^13^C NMR (DMSO-d_6_, 100 MHz, *δ*: 201.56, 177.13, 156.89, 152.53, 138.82, 135.12, 127.35, 36.88, 31.14) spectrometry (Fig. 4B). The ^13^C spectrum showed that there are nine carbon atoms in the SP compound, and each type of carbon atom in the molecule is represented by a peak (Fig. 4D). The NMR data for SP are consistent with those in a previous report by Wang *et al.*^23^.

### Batch and fed-batch biotransformations to SP

**Figure 5:**
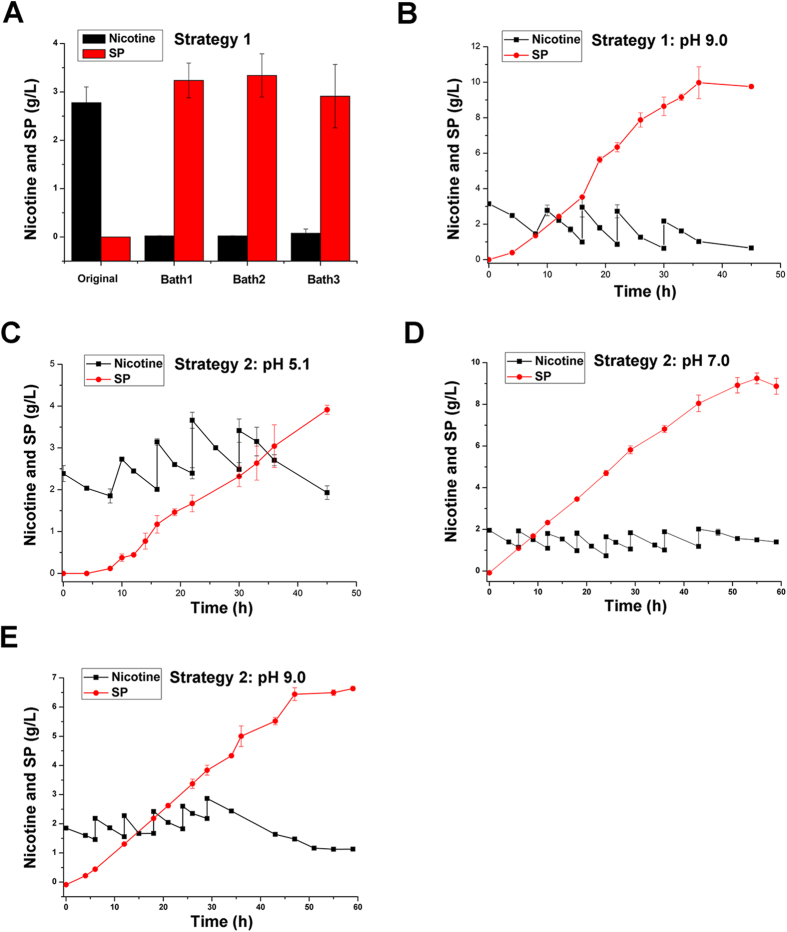
Time course of batch and fed-batch biotransformations by *P. putida* S16dspm. (**A**) For batch biotransformation of aqueous nicotine solution, the biocatalysts were reused three times. Nicotine (black) and SP (red) concentration were analyzed by HPLC at the end of each batch reaction. (**B**) Time course of fed-batch biotransformation from aqueous nicotine solution by *P. putida* S16dspm at pH 9.0, 24 °C, and 5.6 mg/ml DCW resting cells. Nicotine (■); SP (●). (**C–E**) Time course of fed-batch biotransformation by *P. putida* S16dspm from crude tobacco extract at pH 5.1 (**C**), pH 7.0 (**D**), and pH 9.0 (**E**). The fed-batch biotransformation from crude tobacco extract were performed at different pH conditions. Nicotine (■); SP (●).

For batch biotransformation of nicotine to SP, the biocatalyst was reused three times, and each time, the initial nicotine concentration was 3.0 g/L. The SP concentrations with the conversion yields on nicotine were 3.2 ± 0.35 g/L (97.0%), 3.3 ± 0.45 g/L (100.0%), and 2.9 ± 0.65 (87.9%), respectively. The results showed that whole cells of the strain S16dspm could be reused and that, in these subsequent uses, they had almost the same efficient catalytic capabilities as in their first use (Fig. 5A). For fed-batch biotransformation of strategy A, the substrate nicotine was fed intermittently to maintain a substrate concentration above 1 g/L. The time courses of nicotine feeding and SP production are shown in Fig. 5B. SP product was efficiently accumulated after a reaction time of 20 h; the final concentration of SP transformed from nicotine was 9.8 ± 0.11 g/L, with a conversion yield of 83.8% for 45 h.

When crude tobacco extract was used as the substrate in fed-batch biotransformation, the optimal pH for the reaction was 7.0 and the final SP concentration was 8.9 ± 0.38 g/L with a conversion yield of 89.9% (Fig. 5D). Compared to the concentrations produced when using a pH of 7.0 or 9.0, the SP concentration was lowest at pH 5.1 (initial pH value of crude tobacco leaf powder extract): 3.9 ± 0.11 g/L with a yield on nicotine of 54.9% for 46 h (Fig. 5C). The SP concentration obtained using crude tobacco extract was 6.6 ± 0.07 g/L with a yield on nicotine of 85.7% for 59 h (Fig. 5E), which was lower than that obtained using aqueous nicotine solution at pH 9.0 (Fig. 5B).

### Function of the *pps_3984* gene on nicotine degradation in strain S16

**Figure 6:**
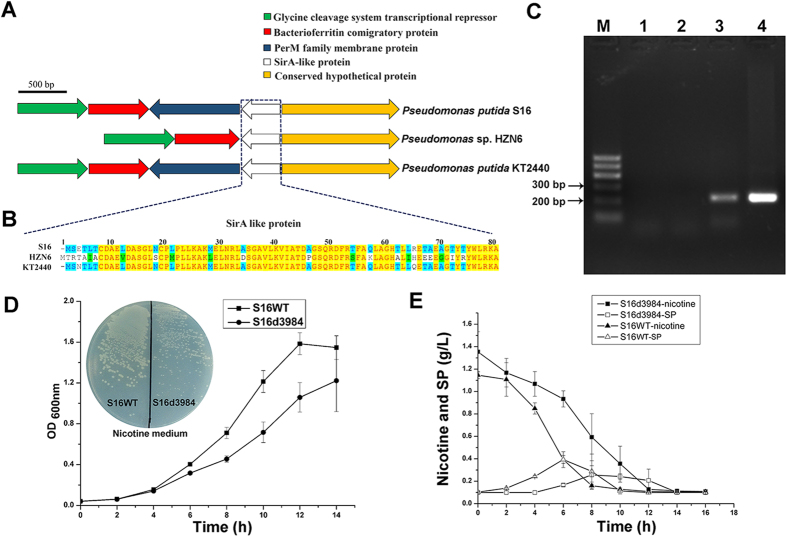
Characteristics of *pps_3984* gene in *P. putida* S16. (**A**) The *sirA* like gene clusters in *P. putida* S16 (GenBank no. NC_015733), *Pseudomonas* sp. HZN6 (GenBank no. HQ832741), and *P. putida* KT2440 (GenBank no. NC_002947). Green–glycine cleavage system transcriptional repressor. Red–bacterioferritin comigratory protein. Blue–PerM family membrane protein. White–SirA-like protein. Yellow–conserved hypothetical protein. (**B**) Multiply sequence alignment of SirA like proteins from *P. putida* S16 (GenBank no. WP_004375233), *Pseudomonas* sp. HZN6 (GenBank no. AEK25019), and *P. putida* KT2440 (GenBank no. NP_743393) using Vector NTI software. Identical conservative sites are highlighted in yellow, comparatively conservative sites are highlighted in blue and green. (**C**) Semi-quantitative RT-PCR analysis of *pps_3984* gene in *P. putida* S16. Pure water (lane 1), total RNA (lane 2), total cDNA (lane 3), and genome (lane 4) from S16 cells grown in nicotine medium were used as templates for PCR with oligonucleotide pairs of *pps_3984* gene. Lane M was DNA marker. (**D**) Growth curves of wild type *P. putida* S16 (S16WT) (■) and the *pps_3984* gene disrupted mutant S16d3984 (●) in nicotine medium with nicotine as the sole carbon and nitrogen source, and S16WT and S16d3984 grown on nicotine plate. (**E**) HPLC analysis of changes of nicotine and SP concentration by culturing S16WT and S16d3984 in nicotine medium. Nicotine degradation by S16d3984 (■). SP metabolism by S16d3984 (□). Nicotine degradation by S16WT (▲). SP metabolism by S16WT (Δ).

The biocatalyst S16dspm in this work lost its ability to degrade metabolite SP^25^. Interestingly, Qiu *et al.* reported that when the *sirA2* (*sirA* like) gene was disrupted in *Pseudomonas* sp. HZN6, the *sirA2*-disrupted mutant lost the ability to degrade SP^28^. In *P. putida* S16, a *sirA-*like gene (*pps_3984* gene) may also exist, located in a similar gene cluster to that of *sirA2*/*sirA* in *Pseudomonas* sp. HZN6 and *P. putida* KT2440 (Fig. 6A). The pps_3984 protein shows 76% and 97% similarity in amino acid sequence identity to SirA2 protein in *Pseudomonas* sp. HZN6 and SirA protein in *P. putida* KT2440, respectively (Fig. 6B). Semi-quantitative reverse transcription polymerase chain reaction (RT-PCR) analysis of *pps_3984* showed that this gene was transcribed when strain S16 was grown in nicotine medium (Fig. 6C). However, the mutant of strain S16 with *pps_3984* gene deletion, S16d3984, could also grow in nicotine medium (Fig. 6D). This mutant strain did not lose the ability of nicotine and SP degradation and had no ability to accumulate metabolite SP (Fig. 6E). These results show that *pps_3984* gene is not crucial in the nicotine degradation of strain S16.

## Discussion

Tobacco wastes containing alkaloids are intractable wastes. In recent years, the nicotine metabolism of *Pseudomonas* strains has been studied in depth, especially in *P. putida* S16 and *Pseudomonas* sp. HZN6^22^,^28^. These strains, with high nicotine degrading efficiency, have potential applications in tobacco waste treatment. Meanwhile, biotransformation by means of engineered microbial cells makes a possibility in resource recovery of tobacco wastes with high nicotine content^12^. In early times, 6-hydroxy-(*S*)-nicotine (productivity, 30 g/L) and 6-hydroxy-3-succinoylpyridine (productivity, 15 g/L) (HSP) were produced by fermentation processes using *Arthrobacter oxydans* NRRL-B-3603 and *Pseudomonas* sp. DSM8653, respectively^16^. In addition, *P. putida* S16 and its derivative P-HSP were developed for the production of HSP in our previous work, and the productivity of HSP could reach 16.3 g/L^13^,^27^. In this paper, genetically engineered strain S16dspm was used to produce a new nicotine analogue 3-succinoyl-pyridine (SP) with a productivity of 9.8 g/L. Over 7000 exciting drugs are pyridine derivatives in the pharmaceutical industry, and the selective functionalization of the pyridine ring is difficult to control by chemical means^17^,^18^,^19^. So the production of simple molecular pyridine compounds, such as SP, HSP or 2,5-dihydroxypyridine (2,5-DHP), by engineered biocatalyst will provide abundant starting materials for the synthesis of bioactivity pyridine drugs.

**Figure 7:**
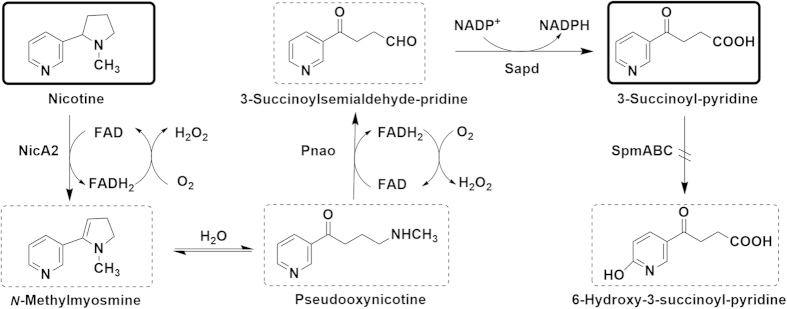
Intermediates and putative cofactors between nicotine and the product SP in *P. putida* S16dspm. Biotransformation from nicotine to SP was catalyzed by multi-enzymes: NicA2, nicotine oxido-reductase; Pnao, pseudooxynicotine amine oxidase; Sapd, 3-succinoylsemialdehyde-pyridine dehydrogenase; SpmABC, SP monoxygenase. Cofactors involved in the reaction between nicotine and pseudooxynicotine were suggested to be FAD/FADH_2_ according to reaction catalyzed by 6-hydroxy-L-nicotine oxidase^30^. Cofactors involved in the reaction from pseudooxynicotine to SP were suggested to be FAD/FADH_2_ and NADP^+^/NADPH according to similar reactions catalyzed by PNAO and SAPD in *Pseudomonas* sp. HZN6^31^.

The advantage of whole-cell biocatalysts is that energy can be provided and cofactors can be recycled when redox reactions are involved^17^,^29^. The biotransformation from nicotine to SP in S16dspm needs multi-enzymatic reactions, and the putative cofactors involved were shown in Fig. 7 30,31. The reaction from nicotine to SP didn’t consume energy, and the putative cofactors FAD/FADH_2_ involved in the reaction from nicotine to 3-succinoylsemialdehyde-pyridine were self-regeneration under aerobic conditions^29^. The last step of reaction from 3-succinoylsemialdehyde-pyridine to the product SP was catalyzed by enzyme Sapd with cofactors NADP^+^/NADPH. However, the Sapd was not essential for nicotine metabolism in S16 because the mutant strain S16dsapd with the deletion of *sapd* gene could still use nicotine to grow. The most reasonable explanation was that 3-succinoylsemialdehyde-pyridine could be easily oxidized to SP by other aldehyde dehydrogenases in the whole cell biocatalysts of mutant strain^25^,^31^. Thus, these facts could explain why the biotransformation in this work using the whole cells S16dspm as biocatalysts was sustainable.

The engineered strain S16dspm achieved high accumulation of SP from both aqueous nicotine and crude tobacco powder extract at the optimal conditions. To test the biocatalytic capability of S16dspm, we explored two strategies. Strategy A: the aqueous nicotine solution extracted from the tobacco leaves was used as substrate (Fig. 2 left). Compared with crude suspension of the tobacco leaves, the reaction solution was simple when the reaction substrate was aqueous nicotine solution, and the optimal pH value for SP production was 9.0 (Fig. 3E), the same as that for HSP production^27^. When the bioconversion reaction system is uncomplicated, the alkaline condition is conducive to SP formation due to its property as a weak acid. Strategy B: the biotransformation could be performed directly in crude tobacco extract in an aqueous solution (Fig. 2 right). However, the initial pH value of crude tobacco extract was 5.1, and as shown in Fig. 5C, this condition was not suitable for SP production. Intriguingly, the SP concentration obtained at pH 9.0 was not the maximum concentration, and it was lower than that obtained at pH 7.0 (Fig. 5D,E) when crude tobacco extract was used as the substrate. This may be due to the fact that crude tobacco extract consists of complex components, including pectins, tannins and alkaloids which can make the reaction mixture become muddy under the alkaline condition and this could inhibit biocatalyst activity. Moreover, SP was isolated from the reaction solution easily in strategy A due to its simple component reaction system. However, when the transformation was carried out in crude tobacco extract using pure water as a solvent in strategy B, the isolation and purification SP-compound crystals remained to be investigated, and would provide a greener approach of SP production than strategy A due to its use of an organic solvent.

In summary, the efficient and specific biocatalyst could be easily prepared using the reusable whole cells of S16dspm. Under the optimal biotransformation process, the isolation yield of crystal compound SP on nicotine was 54.2%. Additionally, this production process for SP reveals that *spmA* gene is crucial in SP degradation of strain S16 rather than the *pps_3984* gene, a *sirA*-like gene. Resource recovery of tobacco waste remains a long-term challenge, and this study thus offers a useful strategy to accomplish the recovery of discarded tobacco wastes.

## Methods

### Chemicals

l-(−)-Nicotine was obtained from Fluka Chemie GmbH (Switzerland). SP was obtained from Toronto Research Chemicals (Canada). All other reagents used were of analytical grade.

### Bacterial strains, plasmids, and culture conditions

*Pseudomonas putida* S16 was isolated from soil and identified as a nicotine-degrading strain as previously decribed^22^. Strain S16 was deposited at Deutsche Sammlung von Mikroorganismen und Zellkulturen with in Gottingen (Germany) under DSM No. 28022. The mutant strain *P. putida* S16dspm was derived from strain S16 in our previous sutudy^25^. The bacterium S16dspm was cultivated in LB medium or glycerin medium containing 1 g/L nicotine at 30 °C.

### Construction of mutant strain S16d3984 and semi-quantitative RT-PCR analysis of *pps_3984* gene

The disruption of gene *pps_3984* was performed using suicide plasmid pK18mob according to the previous methods^25^,^27^. The primers pps_3984F (5′- AGCTAAGCTTCTGTGACGCCGAACTGGAC-3′, *Hind*III) and pps_3984R (5′- GCGAATTCGTGTAGGTACCGGCCTCGGC-3′, *EcoR*I) were used in this work. Semi-quantitative RT-PCR analysis of pps_3984 gene was performed as previously described^32^.

Analysis of the homologous gene cluster in *P. putida* S16, *P. putida* KT2440 and *Pseudomonas* sp. HZN6 and sequence similarity of amino acid were performed by Vector NTI software.

### Determination of nicotine content in discarded tobacco leaves

A batch of discarded tobacco leaves (the tobacco waste) was obtained from Henan province, China. Dried, pulverized, and sieved tobacco powder was prepared for nicotine extraction. Exactly 100 ml of methanol-0.1 M NaOH (1:1, [vol/vol]) were added to 0.5 g tobacco powder, and then the mixture was sonicated for 1 h. After centrifugation at 12,000 rpm for 2 min, this suspension was diluted with twice volume of anhydrous ethanol and prepared for HPLC analysis.

### Extraction of nicotine from discarded tobacco leaves

Strategy A (Fig. 2 right): in order to obtain aqueous nicotine solution, aqueous extracts of tobacco leaves (the tobacco waste) were obtained after step 1, and then the chloroform (1/5 volume) was added to the crude tobacco extract to obtain nicotine in organic phase. Ultimately, back-extraction was performed by dilute sulfuric acid (pH ≤ 4) to get nicotine into aqueous phase.

Strategy B (Fig. 2 left): in order to obtain crude suspension of the tobacco leaves, dried tobacco powder (1 part) was suspended in distilled water (12 parts), and the mixture was left at 60 °C temp with stirring for 200 minutes, then the filter residue was removed to get the crude tobacco extract.

### Transformation of nicotine to SP using S16dspm resting cells

Mutant strain S16dspm was cultured at 30 °C in a rotary shaker at 200 rpm in LB medium or glycerin medium. In mid-exponential phase, cells were harvested by centrifugation at 4,500 rpm for 20 min at 4 °C, then washed with 0.1 M PBS for the first time and with distilled water for twice. The transformation reactions were carried out in a 500-ml flask containing 5.6 mg/ml DCW of resting cells (OD_620nm_ ~ 10), 3–15 g/L nicotine and deionized water, with shaking at 120 rpm at 24–42 °C.

Culture temperature, pH, nicotine content and cellular integrity were studied to identify the optimal conditions for SP production. To identify the optimal temperature for the SP production, reactions were conducted in deionized water containing 3 g/L nicotine at 24 °C, 30 °C, 37 °C, and 42 °C, respectively, with the initial pH 8.0. The optimal pH was identified among pH values of 6.0, 7.0, 8.0 and 9.0. The pH was adjusted using 0.5 M NaOH. Under the optimal temperature and pH condition, the effect of initial nicotine content (3.0, 5.0, 10.0, or 15.0 g/L) was investigated. To evaluate whether the cellular integrity was necessary for the reaction, nicotine transformation was compared between whole-cell and cell-free conditions. The subsequent work was carried out under optimized conditions.

### Batch and fed-batch biotransformations

The biotransformations to SP from aqueous nicotine solution or crude suspension of the leaves powder were conducted in a 1-L conical flask containing 300 ml reaction solution under optimized conditions. For batch transformation, the biocatalyst was collected after the reaction for 8 hours, and reused for another 8 hours. For fed-batch transformation, nicotine was fed at the desired time. Specifically, the reactions in crude leaves powder extract were carried out under different conditions of pH 5.1, pH 7.0, and pH 9.0, respectively.

### Sample preparation and analytical methods

Samples of resting-cell reactions were obtained at desired time, and then twice volume of ethanol was added to stop the reaction. The supernatant obtained after centrifugation at 12,000 rpm for 2 min was filtered by 0.22 μm polyamide filter before quantitative analysis by HPLC. HPLC was performed as previously described^13^.

TLC was used to analyze the metabolite SP. The TLC experiment was performed on 0.2 ± 0.03 mm silica gel (HSGF 254, YJY, Yantai, China) with chloroform-ethanol-methanol-0.5 M NaOH (30:15:2:1.5 [vol/vol]) as mobile phase. The spots of nicotine and SP were shown under UV light (254 nm), or detected by incubating it in iodine vapor.

### Isolation and identification of metabolite SP

After resting-cell incubation with nicotine for ~6 h, the reaction was stopped by removing cells via centrifugation at 8,000 rpm for 10 min at 4 °C. Then the supernatant was evaporated at 50 °C under reduced pressure. The residual liquid was adjusted to pH 3 and then filtration and drying were performed to obtain the white precipitate SP. To identify the structure of metabolite SP, electrospray ionization (ESI)-MS and NMR were performed as previous reports^23^.

## Additional Information

**How to cite this article**: Wang, W. *et al.* Sustainable production of valuable compound 3-succinoyl-pyridine by genetically engineering *Pseudomonas putida* using the tobacco waste. *Sci. Rep.* **5**, 16411; doi: 10.1038/srep16411 (2015).

## Figures and Tables

**Figure 1:**
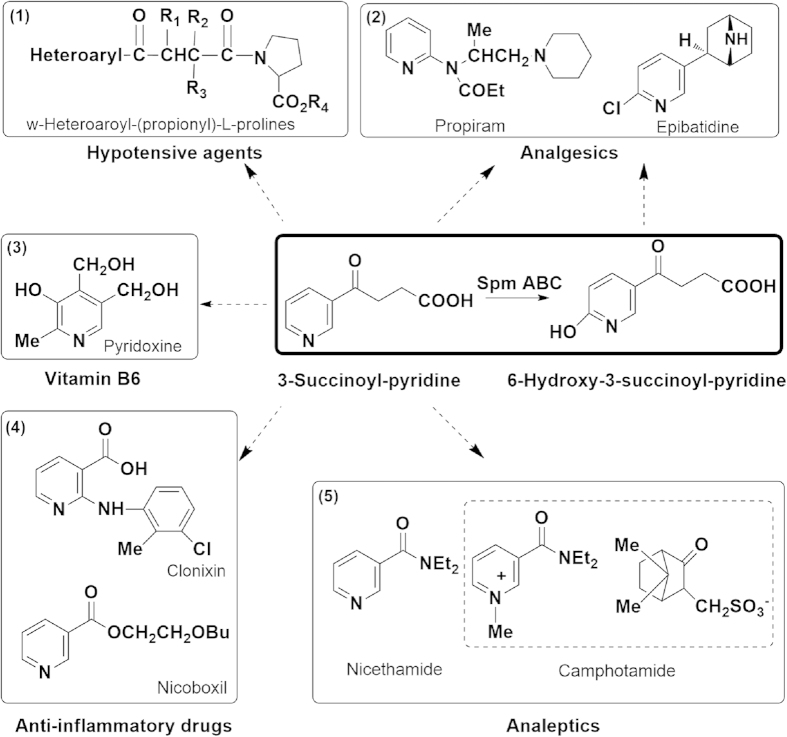
The hypotensive agents and typical pyridine drugs that may be derived from 3-succinoyl-pyridine (SP). Bold-solid lined box: SP is converted to 6-hydroxy-3-succinoyl-pyridine (HSP) by SP monoxygenase (SpmABC) in *P. putida* S16^25^. Solid lined box: (**1**) The hypotensive agent derived from SP, ω-heteroaroyl-(propionyl)-L-prolines^20^. (**2**) Analgesics derived from SP or HSP, propiram and epibatidine^17^,^18^. (**3**) Bioactivity pyridine derivative: vitamin B6 (pyridoxine)^19^. (**4**) Typical pyridine drugs: anti-inflammaroty drugs (clonixin, nicoboxil) and, (**5**) analeptics (nicethamide, camphotamide (dash line box))^18^.

**Table 1:** Determination of nicotine content in discarded tobacco leaves.

**Sample No**	**1**	**2**	**3**
Mass of tobacco powder (g)	0.528	0.507	0.508
Nicotine content of tobacco extraction (g/L)	0.162	0.157	0.158
Nicotine content of tobacco leaves (%)	3.07	3.10	3.11
Average content of nicotine in tobacco leaves (%)	3.09 ± 0.02		

## References

[b1] Davis R. M., Wakefield M., Amos A. & Gupta P. C. The hitchhiker’s guide to tobacco control: a global assessment of harms, remedies, and controversies. Annu. Rev. Public Health 28, 171–194 (2007). 17367285 10.1146/annurev.publhealth.28.021406.144033

[b2] Zhang Y. D., Luo C. R., Wang H. L. & Lu G. F. Advances in microbial degradation of nicotine and its application. Tob. Sci. Technol. 197, 3–7 (2003).

[b3] Mayer B. How much nicotine kills a human? Tracing back the generally accepted lethal dose to dubious self-experiments in the nineteenth century. Arch. Toxicol. 88, 5–7 (2014). 24091634 10.1007/s00204-013-1127-0 PMC3880486

[b4] Dorgan C. A. Statistical record of the environment, 3rd ed. (Gale Research Incorporated, 1995).

[b5] Slack R. J., Gronow J. R. & Voulvoulis N. Household hazardous waste in municipal landfills: contaminants in leachate. Sci. Total Eenviron. 337, 119–137 (2005). 10.1016/j.scitotenv.2004.07.002 15626384

[b6] Schwarzbauer J., Heim S., Brinker S. & Littke R. Occurrence and alteration of organic contaminants in seepage and leakage water from a waste deposit landfill. Water Res. 36, 2275–2287 (2002). 12108720 10.1016/s0043-1354(01)00452-3

[b7] Meher K. K., Panchwagh A. M., Rangrass S. & Gollakota K. G. Biomethanation of tobacco waste. Environ. Pollut. 90, 199–202 (1995). 15091486 10.1016/0269-7491(94)00107-o

[b8] Briški F., Horgas N., Vuković M. & Gomzi Z. Aerobic composting of tobacco industry solid waste—simulation of the process. Clean Techn. Environ. Policy 5, 295–301 (2003).

[b9] Piotrowska-Cyplik A., Olejnik A., Cyplik P., Dach J. & Czarnecki Z. The kinetics of nicotine degradation, enzyme activities and genotoxic potential in the characterization of tobacco waste composting. Bioresour. Technol. 100, 5037–5044 (2009). 19546002 10.1016/j.biortech.2009.05.053

[b10] Gurusamy R. & Natarajan S. Current status on biochemistry and molecular biology of microbial degradation of nicotine. Scientific World Journal 2013, e125385 (2013). 10.1155/2013/125385 PMC3891541 24470788

[b11] Kaiser J. P., Feng Y. C. & Bollag J. M. Microbial metabolism of pyridine, quinoline, acridine, and their derivatives under aerobic and anaerobic conditions. Microbiol Rev. 60, 483–498 (1996). 8840783 10.1128/mr.60.3.483-498.1996 PMC239453

[b12] Iranpour R. *et al.* Environmental engineering: energy value of replacing waste disposal with resource recovery. Science 285, 706–711 (1999). 10426987 10.1126/science.285.5428.706

[b13] Wang S. N. *et al.* “Green” route to 6-hydroxy-3-succinoyl-pyridine from (S)-nicotine of tobacco waste by whole cells of a *Pseudomonas* sp. Environ. Sci. Technol. 39, 6877–6880 (2005). 16190252 10.1021/es0500759

[b14] De Lucas A., Cañizares P., García M. A., Gomez J. & Rodriguez J. F. Recovery of nicotine from aqueous extracts of tobacco wastes by an H^+^-form strong-acid ion exchanger. Ind. Eng. Chem. Res. 37, 4783–4791 (1998).

[b15] Rincon J. *et al.* Preliminary study on the supercritical carbon dioxide extraction of nicotine from tobacco wastes. Sep. Sci. Technol. 33, 411–423 (1998).

[b16] Roduit J. P., Wellig A. & Kiener A. Renewable functionalized pyridines derived from microbial metabolites of the alkaloid (S)-nicotine. Heterocycles 46, 1687–1702 (1997).

[b17] Schmid A. *et al.* Industrial biocatalysis today and tomorrow. Nature 409, 258–268 (2001). 11196655 10.1038/35051736

[b18] Lukevits E. Pyridine derivatives in the drug arsenal (150 years of pyridine chemistry). Chem. Heterocycl. Compd. 31, 639–650 (1995).

[b19] Henry G. D. De novo synthesis of substituted pyridines. Tetrahedron 60, 6043–6061 (2004).

[b20] McEvoy F. J., Wright W. B., Birnberg G. H. & Albright J. D., inventors; American Cyanamid Company, assignee. ω-Heteroaroyl (propionyl or butyryl)-L-prolines. United States patent US 4, 299, 769. 1981 Nov 10.

[b21] Brandsch R. Microbiology and biochemistry of nicotine degradation. Appl. Microbiol. Biotechnol. 69, 493–498 (2006). 16333621 10.1007/s00253-005-0226-0

[b22] Wang S. N. *et al.* Biodegradation and detoxification of nicotine in tobacco solid waste by a *Pseudomonas* sp. Biotechnol. Lett. 26, 1493–1496 (2004). 15604785 10.1023/B:BILE.0000044450.16235.65

[b23] Wang S. N., Liu Z., Tang H. Z., Meng J. & Xu P. Characterization of environmentally friendly nicotine degradation by *Pseudomonas putida* biotype A strain S16. Microbiology 153, 1556–1565 (2007). 17464070 10.1099/mic.0.2006/005223-0

[b24] Tang H. Z. *et al.* Genomic analysis of *Pseudomonas putida*: genes in a genome island are crucial for nicotine degradation. Sci. Rep. 2, 377 (2012). 22530095 10.1038/srep00377 PMC3332521

[b25] Tang H. Z. *et al.* Systematic unraveling of the unsolved pathway of nicotine degradation in Pseudomonas. PLoS Genet. 9, e1003923 (2013). 24204321 10.1371/journal.pgen.1003923 PMC3812094

[b26] Schoemaker H. E., Mink D. & Wubbolts M. G. Dispelling the myths–biocatalysis in industrial synthesis. Science 299, 1694–1697 (2003). 12637735 10.1126/science.1079237

[b27] Yu H., Tang H. Z. & Xu P. Green strategy from waste to value-added-chemical production: efficient biosynthesis of 6-hydroxy-3-succinoyl-pyridine by an engineered biocatalyst. Sci. Rep. 4, 5397 (2014). 24953905 10.1038/srep05397 PMC4066252

[b28] Qiu J. G. *et al.* A *sirA*-like gene, *sirA2*, is essential for 3-succinoyl-pyridine metabolism in the newly isolated nicotine-degrading *Pseudomonas* sp. HZN6 strain. Appl. Microbiol. Biotechnol. 92, 1023–1032 (2011). 21637938 10.1007/s00253-011-3353-9

[b29] Zhao H. M. & van der Donk W. A. Regeneration of cofactors for use in biocatalysis. Curr. Opin. Biotechnol. 14, 583–589 (2003). 14662386 10.1016/j.copbio.2003.09.007

[b30] Kachalova G., Decker K., Holt A. & Bartunik H. D. Crystallographic snapshots of the complete reaction cycle of nicotine degradation by an amine oxidase of the monoamine oxidase (MAO) family. Proc. Natl. Acad. Sci. USA 108, 4800–4805 (2011). 21383134 10.1073/pnas.1016684108 PMC3064382

[b31] Qiu J. G. *et al.* Functional identification of two novel genes from *Pseudomonas* sp. strain HZN6 involved in the catabolism of nicotine. Appl. Environ. Microbiol. 78, 2154–2160 (2012). 22267672 10.1128/AEM.07025-11 PMC3302611

[b32] Wang L. J., Tang H. Z., Yu H., Yao Y. X. & Xu P. An unusual repressor controls the expression of a crucial nicotine-degrading gene cluster in *Pseudomonas putida* S16. Mol. Microbiol. 91, 1252–1269 (2014). 24471758 10.1111/mmi.12533

